# Adapted suicide safety plans to address self-harm, suicidal ideation, and suicide behaviours in autistic adults: protocol for a pilot randomised controlled trial

**DOI:** 10.1186/s40814-023-01264-8

**Published:** 2023-02-28

**Authors:** Jacqui Rodgers, Jane Goodwin, Emma Nielsen, Nawaraj Bhattarai, Phil Heslop, Ehsan Kharatikoopaei, Rory C. O’Connor, Emmanuel Ogundimu, Sheena E. Ramsay, Katie Steele, Ellen Townsend, Luke Vale, Emily Walton, Colin Wilson, Sarah Cassidy

**Affiliations:** 1 Population Health Sciences Institute, Newcastle University, Newcastle Upon Tyne, UK; 2 School of Psychology, Institute of Mental Health, University of Nottingham, Nottingham, UK; 3 Health Economics Group, Population Health Science Institute, Newcastle University, Newcastle Upon Tyne, UK; 4 Social Work, Education and Community Wellbeing, Northumbria University, Newcastle Upon Tyne, UK; 5 Department of Anthropology, Durham University, Durham, UK; 6 Suicidal Behaviour Research Lab, Institute of Health & Wellbeing, University of Glasgow, Glasgow, UK; 7 Department of Mathematical Sciences, Durham University, Durham, UK; 8 School of Psychology, Newcastle University, Newcastle Upon Tyne, UK; 9 School of Psychology, University of Nottingham, Nottingham, UK

**Keywords:** Autism, Self-harm, Suicide, Intervention, Safety plan, Pilot trial, Randomised control trial, Cost-effectiveness analysis

## Abstract

**Background:**

Suicide prevention is a national priority for the UK government. Autistic people are at greater risk of experiencing self-harm and suicidal thoughts and behaviours than the general population. Safety plans are widely used in suicide prevention but have not yet been designed with and for autistic people. We developed the first safety plan specifically targeting suicidality in autistic adults: the Autism Adapted Safety Plan (AASP). It consists of a prioritised list of hierarchical steps that can be used prior to or during a crisis to mitigate risk of self-harm and suicidal behaviour. This is a pilot study that aims to assess the feasibility and acceptability of the AASPs and the research processes, including the response rates, potential barriers and reach of AASPs, methods of recruitment, what comprises usual care, and economic evaluation methods/tools.

**Methods:**

This is an external pilot randomised controlled trial of a suicide prevention tool aimed at mitigating the risk of self-harm and suicidal behaviour in autistic adults: AASPs. Participants will be assessed at baseline and followed up 1 month and 6 months later. Assessments include questions about self-harm, suicidality, service use, and their experience of the AASP/taking part in the study. Autistic adults who have a clinical autism diagnosis and self-reported history of self-harm, suicidal thoughts, or suicidal behaviours within the last 6 months will be invited to take part in the study. Informed consent will be obtained. Participants will be recruited via community and third sector services (including community settings, autism charities, and mental health charities). They may also “self-refer” into the study through social media recruitment and word of mouth. Ninety participants will be randomised to either develop an AASP or receive their usual care in a 1:1 ratio.

**Discussion:**

The present study will provide an evaluation of the suitability of the processes that would be undertaken in a larger definitive study, including recruitment, randomisation, methods, questionnaires, outcome measures, treatment, and follow-up assessments.

**Trial registration:**

ISRCTN70594445, Protocol v4: 8/2/22.

**Supplementary Information:**

The online version contains supplementary material available at 10.1186/s40814-023-01264-8.

## Background

Suicide prevention is a national priority for the UK government, and autistic people have recently been identified as a high-risk group in NICE suicide prevention guidelines [1]. Closing the mortality gap between autistic people and the general population is a priority for the Department of Health’s revised 2018 “Think Autism” strategy [2]. Two James Lind Alliance [3] priority setting exercises have highlighted, as an urgent need, research into adapted mental health and suicide prevention interventions for autistic people [4, 5].

Self-harm refers to any act of self-injury or self-poisoning, regardless of suicide intent [6]. Self-harm has traditionally been conceptualised very differently in autism compared to the general population, which has led to this behaviour being overlooked by researchers and clinicians. In autistic people, self-harm has been viewed primarily as a challenging and/or restricted repetitive behaviour characteristic of autism [7, 8] and is often associated with co-occurring intellectual disability [9]. However, in the general population, self-harm is regarded as an indicator of distress and a significant risk marker for later suicide attempts [10]: between 50–60% of those who die by suicide have previously self-harmed [11, 12]. Furthermore, the years of life lost relating to self-harm is estimated to be 30 years [13]. A majority (up to 65%) of autistic adults experience self-harm, as defined in the general population [14, 15], and self-harm is a significant risk marker for suicidal thoughts and behaviours in autistic adults, after controlling for a range of other risk factors (i.e. age, gender, unemployment, mental health problems, and satisfaction with living arrangements) [14]. Autistic people are also significantly more likely to die by suicide than the general population [16, 17]. Identifying and focusing suicide prevention interventions on people who self-harm, regardless of intent, is important for preventing future deaths and a vital element of suicide prevention efforts [18].

Research highlights a lack of appropriate treatment for autistic adults experiencing self-harm, suicidal thoughts, and suicidal behaviours (Camm-Crosbie et al. 2018). Interventions developed for the general population often do not meet the unique needs of autistic people; for example, differences in social communication and camouflaging one’s autistic traits, in order to “fit in” in social situations [19]. Therefore, interventions need to be adapted in order to meet their needs. There is a growing body of evidence for safety planning in the reduction of suicidal behaviour [20] in the general population, but it is not clear whether they are useful in the autistic population. We developed the first safety plan specifically targeting self-harm and suicidality in autistic adults: Autism Adapted Safety Plans (AASP). Safety plans are simple, scalable, and personalisable suicide prevention interventions, with demonstrated effectiveness in a range of clinical interventions [21, 22]. AASPs have the potential for reducing autistic people’s high risk of self-harm and suicide. We plan to conduct an external pilot randomised control trial to assess the feasibility of the AASP for autistic adults.

## Methods/design

### Design

This is an external pilot randomised controlled trial (ISRCTN70594445) of a suicide prevention tool aimed at mitigating the risk of self-harm and suicidal behaviour in autistic adults: AASPs. It is co-produced with autistic people and will focus on an evaluation of the suitability of the processes that would be undertaken in a larger definitive study, including recruitment, randomisation, outcome measures, treatment, and follow-up assessments.

### Aims

In terms of feasibility, we aim to (1) record the proportion of autistic participants who utilise the AASPs; (2) record response rates for completion of outcome measures, follow-up rates, response rates to questionnaires/assessments, and adherence/compliance rates; (3) record time needed to collect and rehearse methods needed to analyse data related to costs and benefits and health impacts; (4) obtain participant and where possible, non-NHS third sector organisations' feedback on suitability and acceptability of AASPs; (5) investigate potential barriers to and reach of the AASPs; (6) obtain feedback from participants and non-NHS third sector organisations on methods of recruitment, randomisation and the proposed outcome measures, possible use of reinforcement activities, research procedures, and data collection methods to inform a definitive trial; and (7) gather information from participants and non-NHS third sector organisations on what comprises usual care to inform a definitive trial. In addition, we will (1) record the number of instances of self-harm and suicidal thoughts and behaviours in a sample of autistic adults (with recent self-harm and/or suicidal thoughts and/or behaviours) in a 6-month period and (2) compare responses to the outcome measures between the intervention and control arm.

### Setting and participants

Up to 90 autistic adults will be recruited via community and non-NHS third sector organisations (including community settings, autism charities, and mental health charities). Autistic adults may self-refer by approaching the research team directly to participate. Autistic adults are eligible if they meet the following criteria: (1) a clinical autism diagnosis; (2) self-reported history of self-harm, suicidal thoughts, or suicidal behaviours within the last 6 months; (3) sufficient spoken English to complete the assessments; and (4) aged 18 + years. Autistic adults are not eligible for study entry if they are currently experiencing psychotic symptoms.

Non-NHS professionals who support autistic adults (e.g. health and social care professionals and charity support workers) will also be recruited through third sector organisations. They will be trained on how to support an autistic person to develop an AASP and along with members of the research team will be available to support those autistic adults randomised to the safety plan arm to develop their plan. The autistic participant will be able to indicate their preference for either a member of the research team or a support worker to assist with the development of their plan.

### Participant identification and recruitment process

The first participant was consented August 11, 2021. Recruitment is planned to continue until October 31, 2022.

Autistic adults will be identified via non-NHS third sector organisations or via self-referral. Non-NHS third sector organisations that have expressed an interest in participation in the study will assist with recruitment in one of two ways, either (a) they will identify potential autistic people accessing their services who meet the inclusion criteria, discuss the study with them, and, if interested, give them a study pack. Interested autistic adults will then complete an expression of interest form that will be sent to the research team. The research team will then contact the potential participants or (b) promote the study to clients through the self-referral route. In the self-referral route, autistic adults may hear about the study through services they are in contact with, via word of mouth, or through promotion on social media. If an autistic person is interested in the study, they will then contact the research team directly. During contact with potential participants, the research team will discuss the study and what participation involves, answer any questions that they may have, and obtain informed consent. Baseline data will then be collected prior to randomisation.

### Measures

The measures recorded as part of the pilot trial are described below, along with the schedule of measurement (Table 1).

**Table 1:** Time points at which data are collected

Procedure	Screening	Baseline	1-month follow-up (F1)	6-month follow-up (F2)
**Autistic adults & professionals**				
Eligibility checklist	X			
Informed consent	X			
Wellbeing plan (to note adaptations, participant safety, and emergency contact)	X			
**Autistic adults only**				
Demographics^a^		X		X
MINI		X		
SITBI		X	X	X
VEQ^b^		X	X	X
SBQ-ASC		X		X
EQ-5D-5L		X		X
Resource utilisation questionnaire		X		X
Time and travel questionnaire		X		X
Randomisation^c^		X		
Acceptability and feasibility semi-structured interview for autistic adults				X
SUS^d^				X
CSQ-8^d^				X
**Professionals only**				
Acceptability and feasibility semi-structured interview for professionals				X

^a^Demographics to include — socio-economic status, employment, housing, access to support, physical health, and education

^b^At baseline, this questionnaire asks about their entire life. At F1, it asks about the past month. At F2, it asks about the past 5 months

^c^Randomisation to take place following completion of baseline assessment

^d^Only completed by participants allocated to the AASP arm

#### Baseline characterisation

- Mental health
  - Mini-International Neuropsychiatric Interview (MINI; 23): The MINI will be used to assess psychiatric status of participants, including suicidality. It is a short structured diagnostic interview. The MINI is described by the authors as brief and inexpensive, clear and easy to administer, and highly sensitive with good specificity and captures current symptomology [23]. As well as providing diagnostic assessment of suicidality and self-harm, it provides information relating to depression, bipolar I and II, panic disorder, agoraphobia, social anxiety, obsessive–compulsive disorder, posttraumatic stress, alcohol/substance use, psychosis, anorexia nervosa, bulimia nervosa, antisocial behaviours, and generalised anxiety.

#### Feasibility and acceptability outcomes

- Acceptability of all aspects of the trial (including outcome measures, acceptability of intervention materials and methods, and any perceived benefits of the AASP) and feasibility (including experience of recruitment and randomisation) are as follows:
  - Bespoke (i.e. developed specifically for the study) acceptability and feasibility semi-structured interview for autistic adults.
  - Bespoke acceptability and feasibility semi-structured interviews with support staff.
  - System Usability Scale (SUS) [24, 25]: Usability of the AASPs for autistic adults will be measured with the SUS. It consists of a 10-item questionnaire with five response options for respondents; from “strongly agree to strongly disagree”. It is easy to administer, can be used on small sample sizes with reliable results, and can validly differentiate between usable and unusable systems. This measure will only be administered to autistic adult participants who developed an AASP.
  - Client Satisfaction Questionnaire-8 (CSQ-8) [26]: This questionnaire is used to assess level of satisfaction with care. It is widely used in mental health settings. Items are scored on a Likert scale from 1 (low satisfaction) to 4 (high satisfaction) with different descriptors for each response point. Total scores range from 8 to 32, with higher scores indicating greater satisfaction. The CSQ-8 has been found to have high internal consistency and concurrent validity in mental health outpatient settings. This measure will only be administered to autistic adult participants who developed an AASP.

Furthermore, to evaluate whether the study is feasible and acceptable and provides sufficient evidence to progress to a definitive trial, we will also record the following:

1. The number of participants who complete the assessments at the 6-month follow-up (primary endpoint)
2. The percentage of participants who rate the usability of the AASPs on the SUS as 68 or above [27], at the primary endpoint
3. The percentage of participants who report satisfaction with the AASP intervention (indicated as a score > 20 on the CSQ-8) at the primary endpoint.
4. Fidelity of delivery to the AASP manual will be undertaken by experts on the delivery of AASPs viewing the session during which the AASP is developed with the autistic adult and rating the session using a bespoke fidelity checklist.

The criterion for progression to be met across sites is that at least 60% of autistic participants approached over the first 4 months of the external pilot consent to be randomised to the study and complete baseline assessments. This will enable us to determine interest from autistic adults in taking part in the study and the potential recruitment rate for a definitive trial. The stop criterion is that if less than 40% of participants randomised attend the AASP sessions, and/or complete the assessments at the primary end point assessment, it is very unlikely that the target could be achieved in a future definitive trial.

#### Secondary research outcomes

- Self-harm with and without intent to die
  - Self-Injurious Thoughts and Behaviours Interview (SITBI) [28]: The SITBI comprises 74 questions and is widely used. There is acceptable evidence in support of its measurement properties in research [28].
- Suicidal thoughts and behaviours
  - Suicidal Behaviours Questionnaire (SBQ-ASC) [29]: The SBQ-ASC will be used to identify participants’ suicidal thoughts and behaviours. It was developed through participatory methods with autistic adults. The measure has good content validity, structural validity, internal consistency, convergent and divergent validity, test–retest validity, sensitivity, and specificity for distinguishing those with or without lifetime experience of suicide attempt [29].
- Life disadvantages
  - Vulnerability Experience Quotient (VEQ) [30]: The VEQ is a 60-item scale which has been developed through participatory methods with autistic adults to reflect adverse life experiences across 10 themes, such as childhood maltreatment, non-suicidal self-injury, bullying, and victimisation as a child or adult and discrimination.
- Health economics
  - EQ-5D-5L [31]: A standardized instrument used as a measure of health-related quality of life. The descriptive system comprises of five dimensions: mobility, self-care, usual activities, pain/discomfort, and anxiety/depression. Each dimension has a 5-level response scale, from “no problems” to “unable to” engage in the activity (mobility, self-care, usual activities) or “no” to “extreme” (pain and anxiety). The response statements are coded between one to five. The digits for the five dimensions can be combined into a 5-digit number that describes the participant’s health state.
  - Bespoke Resource Utilisation Questionnaire: Provides information to capture the support services participants are accessing, that is, “usual care” derived from prior feasibility study work and existing tools (www.DIRUM.org).
  - Bespoke Time and Travel Questionnaire: Provides information to capture travel time and costs related to contacts with healthcare providers derived from prior feasibility study work and existing tools (www.DIRUM.org).

### Suicide prevention intervention

The intervention will be AASPs, adapted in partnership with autistic people and those who support them, compared to usual care. AASP arm participants will still receive their usual care. Safety plans consist of a prioritized list of hierarchical steps that can be used prior to or during a crisis to mitigate risk of self-harm and suicidal behaviour [32]. They involve identification of (1) warning signs, (2) internal coping strategies, (3) social contacts and locations, (4) family members or friends that may offer help or (5) professionals or agencies to help, and (6) how to keep the environment safe. The safety plan can be personalised to the individual’s needs and have been shown to be efficacious in a range of clinical groups.

We have co-produced an AASP template for autistic adults which is available at https://sites.google.com/view/mentalhealthinautism/resources/safety-plan. Then, we engaged with autistic adults, family members of autistic adults, and service providers who support autistic people, where further adaptations were made to improve the accessibility, functionality, and overall experience of developing an AASP. The AASP is flexible (e.g. timing, method of delivery) and personalisable (e.g. colours and font). Adaptations included prompts with visually distinct key information, a section on “what is important to me” rather than “my reasons for living”, and explicitly considering how the individual communicates distress. The AASP also includes an optional resource kit, with tools to identify emotions, scales/pictorial representations, and suggested support services. Our public and patient involvement (PPI) with autistic people and those who support them has identified safety plans as a promising intervention to prevent self-harm and suicide in autistic adults. They are particularly suitable for autistic people due to the concrete steps involved in formulating the plan. Many autistic people do not realise they are in crisis until it is too late, and due to societal barriers and differences in communication style to non-autistic people, it can be difficult to seek help [33, 34]. AASPs may help autistic people identify warning signs of an approaching crisis and provide them with a personalised plan for seeking help. This is the first evaluation of AASPs.

The AASP will be completed with the support of a trained support worker or member of the research team (depending on participant preference and staff availability) in addition to usual care. The support worker may or may not be at a service the participant is already accessing, depending which organisations agree to take part in the study and which support services the autistic adult is accessing. The training was developed in partnership with autistic people and includes the service provider’s role in the study, information about suicide and self-harm in autistic people (including videos from autistic adults about their experiences with mental health), adaptations from standard safety planning, considerations when working with autistic people (e.g. double empathy problem; [35]), opportunities to discuss and practise the AASP, and feedback from people who have completed the AASP. The training is delivered by the research team.

### Strategies to maintain participation in the study

A number of strategies will be utilised to maintain participation in the study. Newsletters designed by researchers and an advisory committee of autistic adults, summarising the current progress of the study and any findings, will be sent to participants and service providers participating in the study. Similarly, cards will be sent to participants, thanking them for their involvement in the study, to encourage continued participation in the study. Additionally, in cases of nonresponses to researchers by participants, prompts via email, text, or call (depending on the participant’s communication preference) will be sent to encourage participation and minimise participant withdrawal. At baseline, contact information for a trusted person will be collected. They can be contacted in the case of nonresponse from a participant.

### Harm

Serious adverse events (SAEs) will be captured for participants from consent until the follow-up assessment at week 24. A SAE is any untoward medical occurrence in a participant that results in death, is life-threatening, requires inpatient hospitalisation, results in significant disability, and consists of congenital anomaly or other important medical event that jeopardises the participant. Occurrences do not have to be caused by or related to the study. Due to the nature of the study, it is likely that participants are at increased risk of self-harm and suicidal behaviour. We will not report repeated self-harm or suicidality unless it meets the above criteria. Serious adverse events are not anticipated in the study; therefore, any event will be classed as unexpected.

If a clear safeguarding concern arises, this will trigger an immediate response. We have considered potential issues related to safeguarding and have built in mitigation of these into the design. All research associates (RAs) and therapists will be fully trained and have enhanced DBS. A risk register will be developed to manage potential adverse events. A principal investigator (PI) will liaise with RAs to check for SAEs and will take appropriate action, and a chief investigator (CI) will immediately review SAEs in accordance with the trial risk assessment and protocol; both will use clinical judgement in assigning seriousness and causality of SAEs. If any such events are identified, they will be reported to the sponsor and to REC. All such events will be logged in a SAE form and recorded in a site file. If a participant is actively suicidal with imminent risk (e.g. a concrete plan), the RA will discuss with the participant and their CI/PI about the person’s suitability for the study and whether withdrawal is appropriate.

Additionally, researcher wellbeing is encouraged due to the sensitive nature of the study. Research staff have been advised to avoid carrying out sensitive tasks at times when regular support may not be available afterwards (e.g. Friday afternoons) and to schedule in “downtime” after completing potentially upsetting tasks. The research team will have regular informal and formal opportunities to debrief.

### Randomisation

Participants will be randomised on a 1:1 basis to receive either the AASP + usual care or usual care, without stratification. Randomisation occurs online through sealed envelope (https://www.sealedenvelope.com), facilitated by an unblinded RA who will inform participants of the outcome. Participants will be aware of their randomisation status due to the nature of the intervention.

### Procedure

The baseline measures will be completed before the participants are randomised to the treatment arms. For participants recruited to AASP + usual care who were recruited via non-NHS third sector organisations, a trained support worker from that organisation will complete the AASP with the participant; the participant can then use the AASP as required. Individuals who self-refer will either be paired with a trained support worker from one of the non-NHS third sector organisations or will complete the AASP with a member of the research team, based on their preference. With permission, we will record sessions during which the AASP is completed to enable us to determine fidelity. Based on feasibility study work, we anticipate that completion of the safety plan will take approximately 1 h. The support workers will inform the RAs at each site of the date of completion of the safety plan for each participant. Data consent, data collection, and the development of the AASP may take place remotely via telephone or video call.

### Data collection and data management

Overall responsibility for data collection lies with the CI. Data collected on paper assessment tools will be entered onto a secure validated data management system at sites. A unique trial number is allocated at recruitment and will be used to identify participants on all paper assessment tools used throughout the duration of the study. No participant identifiable data will leave the study sites. The quality and retention of study data will be the responsibility of the CI. All study data will be retained in accordance with the latest directive on Good Clinical Practice (GCP; 2005/28/EC) and local policy. Staff involved in the conduct of the study, including PIs, Trial Management Group, and RAs, will have access to the site files. Clinical information shall not be released without the written permission of the participant, except as necessary for monitoring and auditing by the sponsor, its designee, regulatory authorities, or the research ethics committee. Secure anonymised electronic data will, however, be released to the research study analysts for analysis. The PI and site staff involved may not disclose of use for any purpose other than the performance of the study, any data, record, or other unpublished, confidential information disclosed to those individuals for the purpose of the study. Prior written agreement from the sponsor or its designee must be obtained for the disclosure of any said confidential information to other parties. Any requests to access the final trial dataset may be considered under the sponsor data-sharing policy.

### Planned analysis

This external pilot RCT is not powered to estimate a target difference in relative effectiveness but rather to address outcomes to estimate the parameters for a future definitive trial. For pilot studies, a sample size of around 70 participants at endpoint, randomised to treatment vs. treatment as usual, has been recommended to provide sufficient precision to estimate parameters for a full definitive study power calculation [36]; therefore, 90 participants will be recruited to account for drop out.

The feasibility and acceptability outcomes will be analysed using qualitative and descriptive statistics based on mean, standard deviation, median, and interquartile range for continuous data. Number of events and the corresponding percentages will be reported for categorical data. Thematic analysis will be conducted on qualitative feasibility and acceptability interview data. As this is a pilot trial, no formal sample size calculation was performed. However, generalised linear models with appropriate distribution for continuous and categorical data will be used to explore associations between the secondary outcomes and other factors including demographic data. A Bayesian model will be used to estimate the posterior probability that an association between an outcome and a confounder is not the same in the AASP intervention and the usual care groups. For example, if the posterior probability is greater than 60%, we would recommend that such factor is adjusted for a future definitive trial. The external pilot trial will also provide estimates of missing data, which will be calculated as the proportion of participants with outcome data. Using cross-tabulation, we would explore whether participants in the intervention group are more or equally likely to report missing outcome data than those in the usual care group. All data will be analysed under the intention to treat the principle; no interim and no sub-group analysis are planned.

### Dissemination plan

Dissemination of the findings will be undertaken in a number of ways. Newsletters summarising the progress and findings will be designed by the research team and autistic advisors and sent to participants and services who have taken part in recruitment, during the study to support retention, and at the end to share findings. These will also be shared on our study website (https://sites.google.com/nihr.ac.uk/safetyplanstudy/home). A dissemination event will be held at each site at the end of the study. The findings will be presented to autistic adults, local professionals, the study steering group, and stakeholders who supported the study (including the advisory committee of autistic adults). The findings will be disseminated to social care providers, including Crisis Resolution and Home Treatment Teams. The autistic members of the steering group, with support from the research team, write a newsletter for service user organisations and present the study findings at appropriate third sector/professional conferences. Reports will be available in accessible non-NHS third sector organisation newsletters. Dissemination will be done via websites (non-NHS third sector organisations and universities) and social media to access a wider audience. The study findings will be disseminated at the national suicide prevention's strategy steering group for inclusion into future progress reports.

## Discussion

Currently, there are no suicide prevention interventions designed with and evaluated for autistic adults, despite an elevate risk of dying by suicide in this group. Working with autistic people, we have co-produced AASPs to address this gap. AASPs are comprised a template of individualised steps that can be used to identify warning signs, coping strategies, contacts for support, how to keep the environment safe, and how it is best to communicate with the individual autistic adult. Time is also dedicated to discussing storage of the plan, how to know when to use the plan, and what to do if the plan feels like it is not helping. If AASPs are found to be feasible and acceptable, they could be used widely in a variety of settings. This has the potential to fill an unmet need, thus reducing the risk of self-harm and suicide and improving wellbeing in autistic adults. Study outcomes will be used to inform an application for a fully powered multisite intervention trial of autistic adults and associated service providers. Should the AASP demonstrate efficacy in a fully powered trial, then a next step would be to consider modifications for autistic people who may require additional support.

## Conclusions

Autistic people are significantly more likely to die by suicide and engage in self-harm than the general population. Identifying suicide prevention techniques is crucial for preventing deaths. AASPs target suicidality in autistic adults by creating hierarchical steps to be used prior to or during a crisis to mitigate the risk of self-harm and suicide. AASPs have the potential for reducing the high risk of self-harm and suicide in autistic people. Studies evaluating AASPs need to be well informed in terms of methods and outcome measures. The findings of this study are expected to inform a definitive trial.

## Supplementary Information

**Additional file 1. **SPIRIT 2013 Checklist: Recommended items to address in a clinical trial protocol and related documents.

## Data Availability

Anonymised datasets used and/or analysed may be available from the corresponding author upon reasonable request.

## Abbreviations

AASP: Autism Adapted Safety Plans
CI: Chief investigator
CSQ-8: Client Satisfaction Questionnaire-8
GCP: Good clinical practice
MINI: Mini-International Neuropsychiatric Interview
NHS: National Health Service
NICE: National Institute for Healthcare and Excellence
PI: Principal investigator
PPI: Public and patient involvement
RA: Research associate
SAE: Serious adverse events
SBQ-ASC: Suicide Behaviours Questionnaire
SITBI: Self-Injurious Thoughts and Behaviours Interview
SUS: System Usability Scale
VEQ: Vulnerability experiences quotient
